# ELF5-Mediated AR Activation Regulates Prostate Cancer Progression

**DOI:** 10.1038/srep42759

**Published:** 2017-03-13

**Authors:** Kai Li, Yongmin Guo, Xiong Yang, Zhihong Zhang, Changwen Zhang, Yong Xu

**Affiliations:** 1 Department of Urology, Tianjin Institute of Urology, Tianjin Medical University Second Hospital, Tianjin 300211, China; 2 Department of Urology, Tianjin Third Central Hospital, Tianjin 300170, China; 3 Department of Anesthesiology, Qilu Hospital of Shandong University, Jinan 250012, China

## Abstract

The transcription factor E74-like factor 5 (ELF5) is a potent antioncogene that can prevent epithelial-mesenchymal transition (EMT) and metastasis in prostate cancer (PCa). However, little is known how it suppress the tumor growth and if it can interact with androgen receptor (AR). In this study, we find that the ELF5 is frequently expressed in AR activated PCa cells, where it binds to AR acting as a physiological partner and negatively regulates its transcriptional activity. In addition, the interaction between ELF5 and AR is androgen-dependent. Downregulation of ELF5 by shRNA increases the expression of AR-response genes and the progression of PCa. Moreover, ELF5 is a AR-dependent gene that its expression can be induced by androgen and suppressed by antiandrogen treatment. Notably, forced reduction of ELF5 in LNCaP cells facilitates the binding of AR to ARE in ELF5 gene and enabling its transcription, so that low level ELF5 can turn up its own expression by the negative feedback loop.

Prostate cancer (PCa) is the most common cancer among men in the Western countries^1^. Androgen has a overwhelming effect on carcinogenesis in prostate by regulating the transcriptional networks of androgen receptor (AR), genomic stability, and gene fusions, so deprivation of androgen has been a standard treatment of PCa^2^. However, the most formidable challenge in the management of PCa is the development of resistance to androgen deprivation therapy (ADT). The main subset of mechanisms of resistance to ADT include overexpression of AR gene, independently active AR spliced variants, dysregulation of its suppressor, and the increased androgen synthesis^3^. Increased evidence suggest that excessively activated A/AR signaling pathway is the key driver of castration resistant prostate cancer (CRPC)^4^,^5^, therefore, new agents targeting A/AR signaling pathway (such as abiraterone and enzalutamide) were approved for patients with metastatic CRPC (mCRPC)^3^,^6^,^7^. Despite recent advances, the molecular mechanism of how A/AR signaling is reactivated and derives mCRPC remains confusing^4^,^8^,^9^. As a transcription factor, AR regulates its target gene expression through interaction with coregulators, including activators and repressors^10^,^11^,^12^. Since targeting A/AR has limited clinical benefits, great deal of interest in AR coregulators targeted therapy arises to improve current therapies^12^,^13^.

E74-like factor 5 (ELF5) is a transcriptional factor and regulates diverse cellular biology including later stages of terminal differentiation of keratinocytes^14^, trophoblast differentiation^15^ and epithelial-mesenchymal transition (EMT) in tumor cells^16^,^17^,^18^. In pre-stage test, we found that knockdown of ELF5 led to increased cell viability, however, the mechanism remains to be explored. Because of the negative regulatory domain of ELF5 has very low affinity to DNA, so ELF5 appears to regulate cellular biology via non-transcriptional way. Herein, our data show that ELF5 emerged as an androgen-regulated tumor-suppressor gene in PCa. Accordingly, we reveal a resistance mechanism to enzalutamide that caused by loss of ELF5, which results from the remission of AR inhibition by ELF5. Moreover, the findings of low expression of ELF5 in mCRPC confirm that loss of ELF5 is supposed to be one of the mechanisms of CRPC.

## Materials and Methods

### Cell culture and treatment

The cell lines VCaP and LNCaP were originally obtained from the ATCC and maintained in DMEM (VCaP) or RPMI 1640 (LNCaP) (Gibco, Shanghai) supplemented with 10% charcoal-stripped fetal bovine serum (CSF, Gibco, Melbourne), 10 nM dihydrotestosterone (DHT) and 1% penicillin/streptomycin or with 10 μM MDV3100 if indicated.

### Western blotting

Immunoblotting was performed as described previously^16^. Proteins were extracted from adherent cells with Radio Immunoprecipitation Assay (RIPA) lysis buffer. Cell lysates were electrophoresis in 10% Bis-Tris Gel, transferred to PVDF membranes, probed with HRP-linked secondary antibodies, and visualized with ECL reagent (Thermo Scientific).

Used antibodies were as follow: ELF5 (Santa Cruz Biotechnology, catalogue no. sc-376737), cPARP (Cell Signaling, catalogue no. 9541), BCL-xl (Cell Signaling, catalogue no. 2762), AR (Santa Cruz Biotechnology, catalogue no. sc-816), PSA (Dako, catalogue no. A0562) and FKBP5 (Abcam, catalogue no. ab2901).

### Cell proliferation assay

For cell proliferation assays, 20,000 VCaP, 40,000 LNcaP and 5,000 PC3 cells per well were seeded in 24-well plates (n = 3), then cells were harvested and counted at the indicated time points by 3-(4,5-dimethylthiazol-2-yl)-2,5-diphenyltetrazolium bromide (MTT). Before assay, medium was changed to serum-free medium containing MTT, 0.5 mg/mL, Sigma). The cells were incubated with MTT for four hours at 37 °C, then the cellular formazan products were dissolved with acidic isopropanol following the absorbance measurement at 595 nm (Beckman Du640B).

For cell cycle flow cytometry assay, 1 × 10^6^ cells were fixed with 70% ethanol at 4 °C for 12 h. After that, cells were centrifuged (1,000× g, 5 min, 4 °C), resuspended in PBS containing 0.05 mg/mL RNase A (Sigma), and incubated at room temperature for 30 min. After washing, cells were stained with 10 mg/mL propidium iodide and filtered with a 60-μm mesh, and 10,000 cells were used to be analyzed by flow cytometry (FACSCalibur, BD Company) with ModFit software (Verity Software House, Inc.).

### Murine prostate tumor xenograft model

Male immunocompromised (nonobese diabetic [NOD]-severe combined immunodeficiency [SCID]) mice were procured from a breeding colony at Chinese Academy of Sciences maintained by our group. About 1 × 10^7^ LNCaP cells suspended in 100 μl of PBS with 50% Matrigel (BD Biosciences) were subcutaneously implanted into the dorsal flank on both sides of the mice. For enzalutamide treatment, once the tumors reached a tangible size (200 mm^3^), the mice were randomized and treated with either 20 μl DMSO with 10 mg·kg^−1^ body weight enzalutamide or 20 μl DMSO without enzalutamide by intraperitonially for five days a week. Tumor volumes were estimated using the formula (π/6)(L × W^2^), where L is length and W is width of tumors. At the end of the studies femur bone marrow, liver and spleen were harvested to determine spontaneous metastasis by measuring human-Alu sequence as previously described^19^. Briefly, genomic DNA from femur bone marrow, liver and lung were prepared followed by quantification of human Alu expression by human Alu-specific fluorescent TaqMan qPCR probes. All procedures involving mice were approved by the University Committee on Use and Care of Animals at the Tianjin Medical University and conformed to all regulatory standards.

### Patient samples and histological staining

Paraffin-embedded tissues of PCa patients were acquired from the Department of Pathology, Tianjin Institute of Urology in compliance with protocols approved by the institutional review board. Informed consent from each patient have been obtained. For staining, paraffin-embedded sections (4 mm) were successively deparaffinized and hydrated in xylene, graded alcohols and water. Antigen retrieval was performed by using 0.01 M citrate. After washing, slides were then incubated with ELF5 antibodies (Santa Cruz Biotechnology, Santa Cruz, CA) at 4 °C for 16 hr, followed by Envision-plus-labeled polymer-conjugated horseradish peroxidase and DAB (Zhong Shan gold bridge, Beijing). Stainings were evaluated and scored by a clinical pathologist (Z.Z) in a blinded fashion. Five random low magnification fields were selected, then the proportion of positive nucleus was determined by ImageJ program.

### BrdU and TUNEL staining

5′-Bromo-2′-deoxyuridine (BrdU, Sigma) was dissolved in double distilled water at 10 mg/ml. On the day before sacrifice, the solution was injected intraperitoneally to mice every 6 h (10 μg/g). For BrdU staining, paraffin-embedded sections were deparaffinized in xylene and hydrated in alcohols and water. For antigen retrieval, slides were bathed in 0.01 M citrate at 95 °C for 10 minutes followed by a 60-min cool down. Slides were then incubated with BrdU antibody and DAB monitoring staining (Zhong Shan gold bridge, Beijing). At the end, slides were counterstained and dehydrated.

For TUNEL staining, the experiment was performed according to manufacturer’s instructions of the In Site Cell Death Detection Kit purchased from Roche. In brief, tissue sections were incubated with proteinase K at 37 °C for 15 min after dewaxing and hydrating. After washing, tissue sections were incubated with TUNEL reaction mixture and the samples were analyzed under fluorescence microscope.

### Transfection, Infection, and Treatment

ELF5 was successfully knocked down using psi-LVRH1P lentiviral vectors from the RNAi consortium shRNA library (purchased from GeneCopoeia, catalogue no. HSH004744-LVRH1P). Stable knockdown was achieved by 1 μg/ml puromycin selection. Knockdown effects were confirmed by western blotting.Elf5 was tagged at the 3′ end with Myc and incorporated into the pReceiver-Lv125 vector (purchased from GeneCopoeia, catalogue no. EX-Z5383-Lv125). Elf5 expression was achieved using 1 μg/ml puromycin.

### Immunoprecipitation

For immunoprecipitation following ectopic expression, proteins were extracted from adherent cells with m-Per buffer (Pierce). After centrifuging, the supernatants were incubated with AR antibody over night and proteins were pulled down by protein A/G agarose (SantaCruz Biotechnology). After washing, beads were dissolved in 30 ul of 2× SDS sample buffer for Western blotting.

### RNA Extraction and RT-qPCR

For cell lines, RNA was extracted using the RNeasy kit (Qiagen). For frozen tumors, RNA was extracted using Trizol (Invitrogen) followed by clean up with RNeasy (Qiagen). cDNA was generated with the Transcriptor First Strand cDNA Synthesis Kit (Roche). qPCRs were performed in triplicate using FastStart Universal SYBR Green Master (Roche) according to manufacturer’s instructions. The target mRNA expression was quantified using the ∆∆C_t_ method and normalized to beta-Actin or GAPDH expression and relative expression was generally plotted.

Primers for RT-qPCR are as follows: AR (F: CCATCTTGTCGTCTTCGGAAATGTTATGAAGC, R: AGCTTCTGGGTTGTCTCCTCAGTGG), FKBP5 (F: CAGATCTCCATGTGCCAGAA, R: CTTG CCCATTGCTTTATTGG), GAPDH (F: TGCACCACCAACTGCTTAGC, R: GGCATGGACTGTGGTCATGAG), PSA (KLK3) (F: GTCTGCGGCGGTGTTCTG, R: TGCCGACCCAGCAAGATC), TMPRSS2 (F: CAGGAGTGTACGGGAATGTGATGGT, R: GATTAGCCGTCTGCCCTCATTTGT) and ERG (F: CGCAGAGTTATCGTGCCAGCAGAT, R: CCATATTCTTTCACCGCCCACTCC).

### Chromatin Immunoprecipitation

LNCaP or LNCaP-shELF5 cells were maintained in steroid depleted media for 4 days. The day prior to CHIP, cells were given fresh indicated media. Fixation and processing were carried out as described previously^20^. Immunoprecipitation was carried out with AR (N20) Antibody or Normal Rabbit IgG.

Primers for CHIP-PCR are as follows: FKBP5 (F: CCCCCTATTTTAATCGGAGTAC, R: TTTTGAAGAGCACAGAACACCCT), PSA (KLK3) (F: TTTCATCCTGGGCGTGTCTC, R: CTCCCCCAGGAGCCCTATAA) and TMPRSS2 (F: GTGAATTCTGAGCCCCCACA, R: GTGAGGAAGTGGTGGGACAC).

### Statistical analysis

Data were generally presented as mean ± SE unless otherwise stated. Student *t* test and One-way ANOVA were used in this study. SPSS (v.19, IBM, USA) was used to assess date. *P* < 0.05 was considered statistically significant.

## Results

### ELF5 Knockdown Promotes Prostate Cancer Cell Growth *In Vitro*

**Figure 1:**
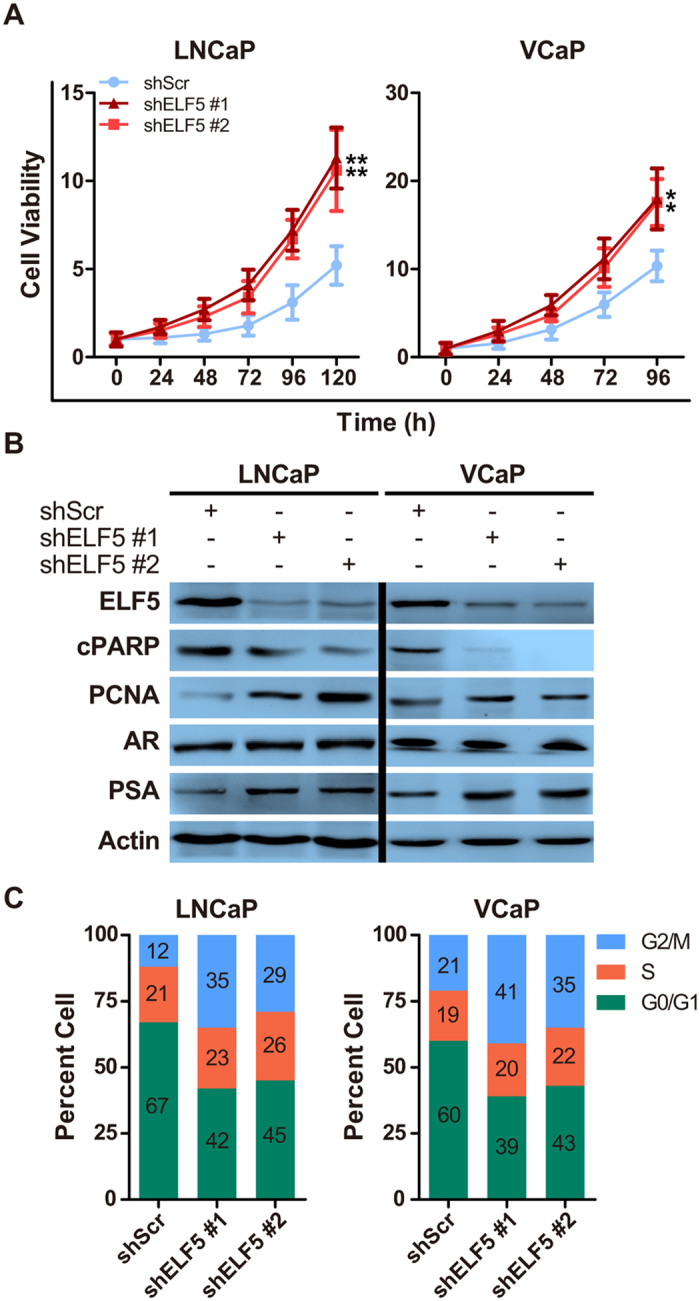
Knockdown of ELF5 Promotes Prostate Cancer Cell Growth *In Vitro*. (**A**) Analysis of LNCaP or VCaP cell growth after downregulation of ELF5 by two different shRNAs. **P < 0.01. (**B**) WB analysis of cPARP, PCNA, AR and PSA proteins obtained from LNCaP or VCaP cells after downregulation of ELF5. (**C**) Cell cycle analysis of LNCaP or VCaP cells after downregulation of ELF5. Data represent three independent experiments.

In the course of investigating the function of ELF5 in PCa progress, we discovered downregulation of ELF5 in stably selected ELF5 knockdown cells (infected by shRNAs for ELF5, shELF5) potently stimulated the viability of human PCa LNCaP and VCaP cells (Fig. 1A). To learn how the loss of ELF5 in cancer cells supports its viability, we assessed proliferation and apoptosis markers in both stably selected LNCaP and VCaP cells infected by shELF5 (LNCaP-shELF5 or VCaP-shELF5) or scramble shRNA (shScr) (LNCaP-shScr or VCaP-shScr) (Fig. 1B). We found PCNA (proliferation marker) increased and cleaved PARP (cPARP, apoptosis marker) decreased in ELF5-knockdown PCa cells (Fig. 1B). To confirm the function of ELF5 in promoting viability, cell cycles evaluated by flow cytometry were conducted. We detected that cells in fraction of G2M stage were significantly increased after the downregulation of ELF5 (Fig. 1C). Altogether, these results demonstrate that loss of ELF5 promotes cell viability via promoting proliferation and suppressing apoptosis in PCa.

### Downregulated ELF5 Increases Xenograft Prostate Tumor Growth and Metastasis

**Figure 2:**
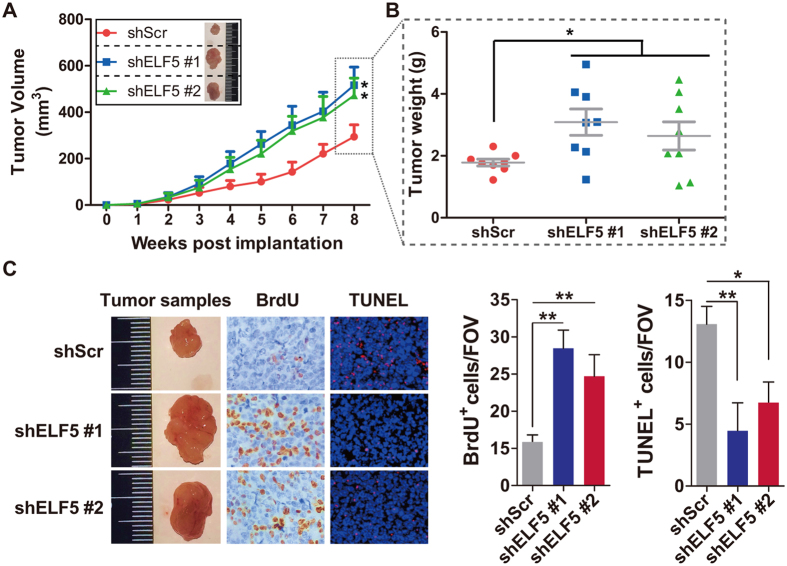
Downregulated ELF5 Increases the development of LNCaP Xenograft. shScr or shELF5 infected LNCaP cells were injected subcutaneously into NOD-SCID mice, with 8 mice per group. (**A,B**) The mean tumor volume and the distribution of individual weight are shown. *P < 0.05. (**C**) Mice were sacrificed at eight weeks post implantation. The tumor samples, BrdU stain, TUNEL stain and the quantity analyses are shown. All images were collected at the same magnification.

To explore the function of ELF5 suppression *in vivo*, we analyzed xenografts of LNCaP prostate cancer cells. As expected, tumors arising from LNCaP-shELF5 grew significantly faster than those arising from LNCaP-shScr cells (Fig. 2A,B). To better understand the result, we excised tumors at 8 weeks after injection and examined their tumor specimens and histology (Fig. 2C). Tumors from mice injected with LNCaP-shELF5 shared more BrdU and less TUNEL expression, indicating their higher proliferation and lower apoptosis (Fig. 2C). In contrast, LNCaP-shScr produced tumors with lower proliferation and higher apoptosis (Fig. 2C).

In our previous study, we had found that downregulated ELF5 could promote migration, invasion and spheres formation in LNCaP cells^16^. Could the phenotype be found *in vivo*? To test whether deceased ELF5 leads to spontaneous metastasis in our LNCaP xenograft model, we isolated femur, liver and spleen from Xenograft-bearing mice and found evidence of increased metastases in mice injected with LNCaP cells infected by shELF5 (Fig. S1).

### ELF5 Weaken Resistance of Prostate Cancer to Enzalutamide

**Figure 3:**
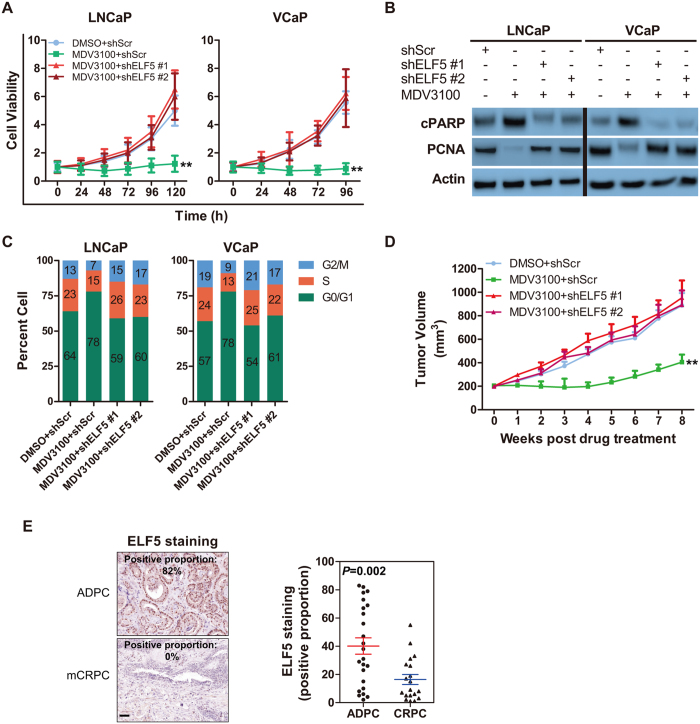
ELF5 Weaken Resistance of Prostate Cancer to Enzalutamide. (**A**) Cell growth analysis of LNCaP or VCaP cells infected by shScr or shELF5 after DMSO or enzalutamide (10 μM for 120 h). **P < 0.01. (**B**) WB analysis of cPARP and PCNA proteins obtained from LNCaP or VCaP cells infected by shScr or shELF5 after DMSO or enzalutamide (10 μM for 120 h). (**C**) Cell cycle analysis of LNCaP or VCaP cells infected by shScr or shELF5 after DMSO or enzalutamide (10 μM for 120 h). Data represent three independent experiments. (**D**) The mean tumor volume of xenografts arose from LNCaP cells infected by shScr or shELF5 after DMSO or enzalutamide. Results are mean volume ± SEM for 8 to 10 mice per group. **P < 0.01 compared with DMSO + shScr group (ANOVA) (**E**) Representative images and quantification of staining proportion for ELF5^16^ in the specimens from 24 androgen-dependent prostate cancers (ADPC) and 19 CRPC with bone metastases (mCRPC). For each specimen, five random regions were evaluated. For each region, the nuclear positive of ELF5 was quantified by using Image J software (1.6.0_24). Data represent the average expression score. p values determined by Wilcoxon’s test. Scale bar, 100 μm.

Enzalutamide (MDV3100) has been the best effective androgen deprivation therapy (ADT) for patients with PCa in an effort to shrink or removal the tumor. Although the treatment usually inhibit tumor growth, some cases are resistant to it. Reviewing above results, we have known decreased ELF5 promotes proliferation and inhibits apoptosis in PCa cell. So we want to known if decreased ELF5 deteriorates or contributes to enzalutamide resistance, we detected downregulation of ELF5 stimulated the viability of LNCaP and VCaP cells treated by enzalutamide (Fig. 3A). More detailed, We found higher PCNA and lower cPARP in ELF5-knockdown cells (Fig. 3B). As expected, we also found more cell fraction in G2M stage after enzalutamide treatment in ELF5-downregulated cancer cells (Fig. 3C). On the contrary, ELF5 overexpression weakened the viability of PC3 cells with or without enzalutamide treatment (Fig. S2A). Additionally, less proliferation and more apoptosis were observed in ELF5-overexpressed PC3 cells with or without enzalutamide treatment (Fig. S2B).

To explore the function of ELF5 suppression on enzalutamide resistance *in vivo*, we analyzed xenografts of LNCaP PCa cells. Consistent with previous results, LNCaP-shScr tumor-bearing mice treat with enzalutamide led to a significant reduction in tumor volume (Fig. 3D)^19^. Whereas, enzalutamide had a less pronounced effect in tumors arising from LNCaP-shELF5 (Fig. 3D). Enzalutamide has been demonstrated to promote metastasis in preclinical model^19^,^21^. To test this in xenograft model arising from LNCaP-shELF5, we isolated femur, liver and spleen from enzalutamide-treated mice, evidence showing that enzalutamide increased metastases in femur, liver and lung (Fig. S3).

To address whether the ELF5 is downregulated in patients with advanced disease, we stained matched sections, which are from a cohort of patients withandrogen dependent PCa (ADPC) and CRPC. Consistent with our experimental models, a significant decrease in ELF5-expressing cells was observed in tumors from patients with CRPC (Fig. 3E). Taken together, these pre-clinical studies suggest that the lower expression of ELF5 can partly balance out the function of enzalutamide. In addition, the use of enzalutamide in clinically localized prostate cancer may potentiate metastasis independent of ELF5 expression.

### ELF5 Regulates Expression of AR-responsive Genes

**Figure 4:**
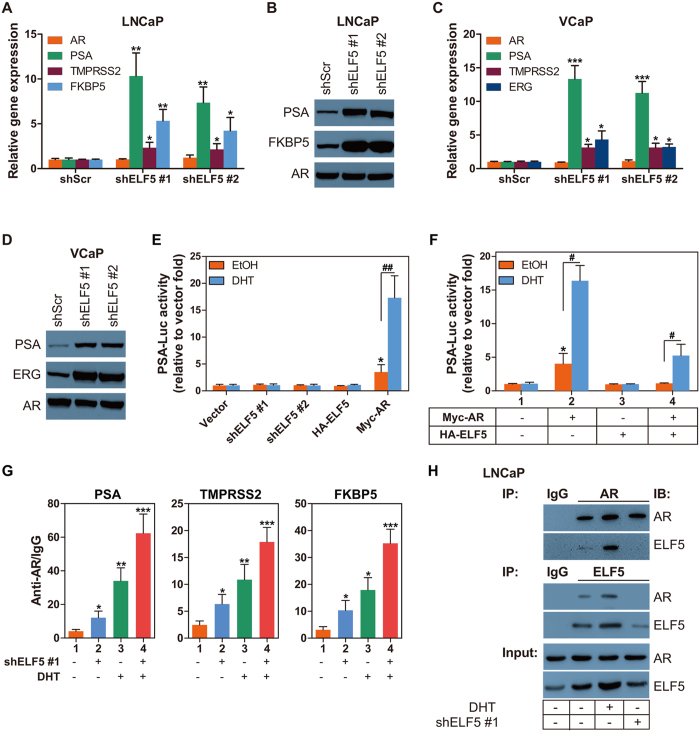
ELF5 Regulates The Expression of AR-responsive Genes. (**A**) Quantitative PCR analysis of AR and AR responsive genes KLK3 (PSA), TMPRSS2 and FKBP5 in LNCaP cells that were transiently transfected with shScr or two different shELF5. qPCR with total RNA was performed at 72 h post transfection. Data are from two independent experiments in triplicate. (**B**) WB analysis of PSA and FKBP5 proteins obtained from LNCaP cells that were transiently transfected with shScr or two different shELF5. WB was performed at 72 h post transfection. (**C**) Quantitative PCR analysis of AR and AR responsive genes KLK3 (PSA), TMPRSS2 and ERG in VCaP cells that were transiently transfected with shScr or two different shELF5. qPCR with total RNA was performed at 72 h post transfection. Data are from two independent experiments in triplicate. (**D**) WB analysis of PSA and FKBP5 proteins obtained from VCaP cells that were transiently transfected with shScr or two different shELF5. WB was performed at 72 h post transfection. (**E,F**) PSA promoter reporter (PSA-Luc) activity assay in LNCaP cells that were transiently transfected with vector, two different shELF5, HA-ELF5 and Myc-AR, followed by EtOH or 10 nM DHT treatment for 48 h. The PSA-Luc activity assay was performed at 72 h post transfection. *compared with line 1, *P* < 0.05; ^#^*P* < 0.05, ^##^*P* < 0.01. (**G**) CHIP was performed with AR-N20 antibody using material prepared from LNCaP cells that were transiently transfected with shScr or shELF5, followed by EtOH or 10 nM DHT treatment for 48 h. Normal rat IgG served as a negative control. Results were analyzed by qPCR with primers targeting potential heat shock elements in human PSA, TMPRSS2 and FKBP5. The experiment was repeated twice. *P < 0.05, **P < 0.01, ***P < 0.001. (**H**) CO-IP and WB analysis of AR and ELF5 proteins in total lysates obtained from LNCaP cells that were transiently transfected with shScr or shELF5, followed by EtOH or 10 nM DHT treatment for 48 h.

In Fig. 1B, we come across downregulated ELF5 led to increased prostate specific antigen (PSA) without influencing the AR expression, which reflect a potentially inhibiting effect of ELF5 on AR transcriptional function. To verify this, quantitative PCR and western blotting are conducted to test other AR responsive genes expression. Results demonstrate that ELF5 downregulation significantly improved the endogenous expression of well-characterized AR-dependent genes PSA (KLK3), FKBP5, ERG and TMPRSS2 at mRNA and protein levels (Fig. 4A–D). As in Fig. 1B, ELF5 expression have no effect on AR itself expression (Fig. 4A–D).

To investigate if ELF5 transcriptionally regulates AR responsive gene expression, we introduced PSA promoter-driven luciferase reporter (p61-Luc) assays. HEK293 cells, having no endogenetic AR or PSA expression, were transiently co-transfected with p61-Luc reporter along with mock (control), HA-ELF5, shELF5 or Myc-AR construct plasmid, followed by treatment with vehicle or DHT. We found that transient expression of AR increased the androgen induction of PSA promoter reporter (PSA-Luc) activity about 16.5 fold compared with mock vector, and 6.5 fold compared with its neighbor group that without DHT treatment (Fig. 4E). We also found that neither solo enhance or silence the expression of ELF5 has direct effect on p61-Lcu reporter activation (Fig. 4E). However, when forced to express accompanied AR, ELF5 exhibited its effects in suppressing p61-Lcu reporter activation (Fig. 4F). So, we guess the suppression of ELF5 on AR responsive genes expression are AR dependent. For this, we conducted chromatin immunoprecipitation (CHIP) following shELF5 and/or DHT treatment. Results proved that ELF5 downregulation considerably maked improvement in the combination of AR and its dependent genes PSA, FKBP5 and TMPRSS2 regardless of DHT treatment (Fig. 4G). Moreover, we found that ELF5 suppressed AR responsive genes expression via binding to AR (Fig. 4H). The interaction between ELF5 and AR is androgen-dependent.

### ELF5 as an AR Response Gene Regulates Itself Expression

**Figure 5:**
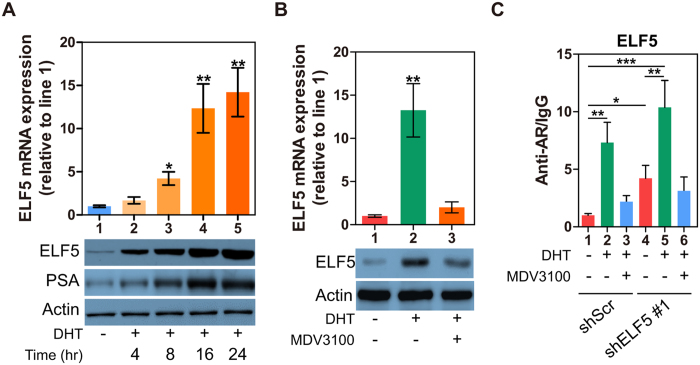
ELF5 as an AR Response Gene Regulates Itself Expression. (**A, top**) qPCR analysis of ELF5 gene expression in LNCaP cells that were treated by different time of 10 nM DHT; (**A, bottom**) WB analysis of ELF5, and PSA proteins in LNCaP cells that were treated by different time of 10 nM DHT. (**B**) qPCR and WB analyses of ELF5 expression in LNCaP cells that were treated by DHT or enzalutamide. (**C**) CHIP was performed with AR-N20 antibody using material prepared from LNCaP cells that were transiently transfected with shScr or shELF5, followed by DHT or enzalutamide treatment for 48 h. Normal rat IgG served as a negative control. Results were analyzed by qPCR with primers targeting potential heat shock elements in human ELF5. The experiment was repeated twice. *P < 0.05, **P < 0.01, ***P < 0.001.

Notably, considerable level of ELF5 expresses in LNCaP, VCaP and 22Rv1 cells, whereas little expresses in PC3 and DU145 cells (Fig. S4A). So, we question whether ELF5 is special for AR positive cells, in other word, if ELF5 is the direct transcriptional target of AR. A bioinformatic search for androgen-response elements (AREs) in ELF5 promoter confirmed that the ELF5 gene contained canonical ARE (Fig. S4B). To determine whether AR directly regulates ELF5 expression in LNCaP cells, we performed qPCR and western blotting following DHT treatment. We found that ELF5 increased after DHT treatment in a time-dependent manner (Fig. 5A). In addition, the increased ELF5 could be inhibited by enzalutamide (Fig. 5B). To confirm this, we performed ChIP using AR-N20 antibody and extracts prepared from LNCaP cells infected by shScr or shELF5. IgG served as a negative control. ELF5 was significantly amplified bound by AR after DHT treatment, and suppressed by enzalutamide (Fig. 5C). Notably, knockdown of ELF5 can significantly increase the binding of ELF5 DNA to AR (Fig. 5C). Together with the effects of AR seen on expression of ELF5, these data suggest that ELF5 can regulate itself through a negative feedback that depend on AR activation.

## Discussion

The major mechanism of resistance to castration is an acquired capability that allows CRPC to survive under the surroundings without sufficient DHT for AR activation and tumor progression. Identifying coregulators (both coactivators and corepressors) that are implicated in the reprogramming of AR function would give us more helpful opportunities for CRPC detection and individually therapeutic intervention. In this study, we demonstrated that ELF5 negatively correlated with the PCa progress, including proliferation and metastasis. After downregulation of ELF5, PCa cells acquired more rapid growth, xenograft tumors arisen from these PCa cells grew faster and more inclined to metastasis. *In vivo*, reduced ELF5 expression can promote tumor growth and induce the most probability of distant metastasis in mice bearing LNCaP xenograft tumors receiving enzalutamide treatment.

Beside that, two significant observations were noted in this study. First, we have demonstrated that an interaction between ELF5 and AR may play a important role in the development of CRPC. We provide convincing evidence that the interaction between ELF5 and AR are androgen independent. Our data also reveal that downregulated ELF5 in LNCaP cells increases AR gathering in the promoter of its response genes, which coincide with augmented expression of AR-responsive genes, independently of androgen exposure, and that the increased expression of ELF5 has the opposite effect. Second, we find that ELF5 is a downstream gene of AR, which has been shown to directly bind to ARE in ELF5 promoter. As a corepressor of AR, ELF5 can regulate its own expression by inhibiting the gathering of AR in the ARE of its promoter.

Previous studies have proposed the primary developmental target of ELF5 is the mammary progenitor cell population^22^. Coupled with estrogen stimulation, ELF5 promotes progenitor cells differentiating into estrogen receptor (ER) negative cells, and ultimately into a mature acinar cell capable of large-scale milk synthesis^22^. Whereas, the same progenitor cell would differentiated into ER positive cell with different functions if the ELF5 is replaced by FOXA1^23^. It is likely that coregulators (e.g. ELF5 and FOXA1) provide the direction of the decision made by the progenitor cell. In this study, our data show that ELF5 has potent effects in suppressing proliferation and inducing apoptosis in LNCaP cells or its xenograft model. In addition to key regulator of mammary gland alveologenesis, ELF5 has also been demonstrated to be a prominent inhibitor of EMT in breast, bladder and prostate cancers^16^,^17^,^18^. Furthermore, it has been proved that ELF5 suppresses EMT by directly repressing the transcription of Snail2 and the transcriptional activity of SMAD3, two master inducers of stem cells and EMT^16^,^18^.

The affinity of enzalutamide to the ligand-binding domain of AR is extraordinary lower than that of DHT^24^. Despite the potent activity of the AR antagonist, clinical studies in patients with CRPC treated with this drug have proved the block is incomplete and the resistance always exists^3^,^25^,^26^. Our data demonstrate that the reduced expression of ELF5 is associated with higher proliferation and lower apoptosis in LNCaP cells and its xenograft model received enzalutamide therapy, suggesting a mechanism for clinical resistance to enzalutamide. Moreover, future investigations are warranted to explore the stability of ELF5 in PCa, so that the therapeutic efficacy of ELF5 can be discovered.

## Additional Information

**How to cite this article**: Li, K. *et al*. ELF5-Mediated AR Activation Regulates Prostate Cancer Progression. *Sci. Rep.* **7**, 42759; doi: 10.1038/srep42759 (2017).

**Publisher's note:** Springer Nature remains neutral with regard to jurisdictional claims in published maps and institutional affiliations.

## Supplementary Material

Supplementary Information
