# Supplementary material for: ELF5-Mediated AR Activation Regulates Prostate Cancer Progression
Source: Sci Rep. 2017 Mar 13;7:42759. doi: 10.1038/srep42759 (PMC5347131; doi:10.1038/srep42759)
Supplement: Supplementary Information [file srep42759-s1.doc]

ELF5-AR Feedback Loop in Regulating Malignant Behavior of Prostate Cancer Cells

Kai Li^1,2^, Yongmin Guo^3^, Xiong Yang^1^, Minghao Zhang^2^, Zhihong Zhang^1^, Changwen Zhang^1^, Yong Xu^1^

1 Department of Urology, Tianjin Institute of Urology, Tianjin Medical University Second Hospital, Tianjin 300211, China.

2 Department of Urology, Tianjin Third Central Hospital, Tianjin 300170, China

3 Department of Anesthesiology, Qilu Hospital of Shandong University, Jinan 250012, China.

Li and Guo contributed equally to this work.


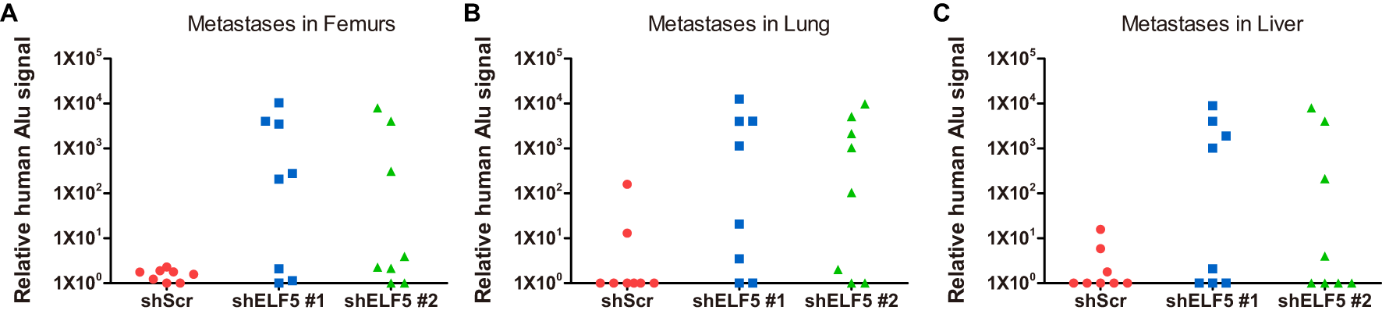


**Figure S1.** ELF5 knockdown leads to spontaneous metastasis. Mice bearing xenografts arose from LNCaP cells, infected by shScr or shELF5 (see also **Figure 2**), were assessed for spontaneous metastasis to bone (femur), lung and liver. To analyze metastasized cells in indicated sites, human Alu sequence was measured in isolated genomic DNA by using qPCR. Results show ELF5 knockdown increases spontaneous metastasis to femur, lung and liver.


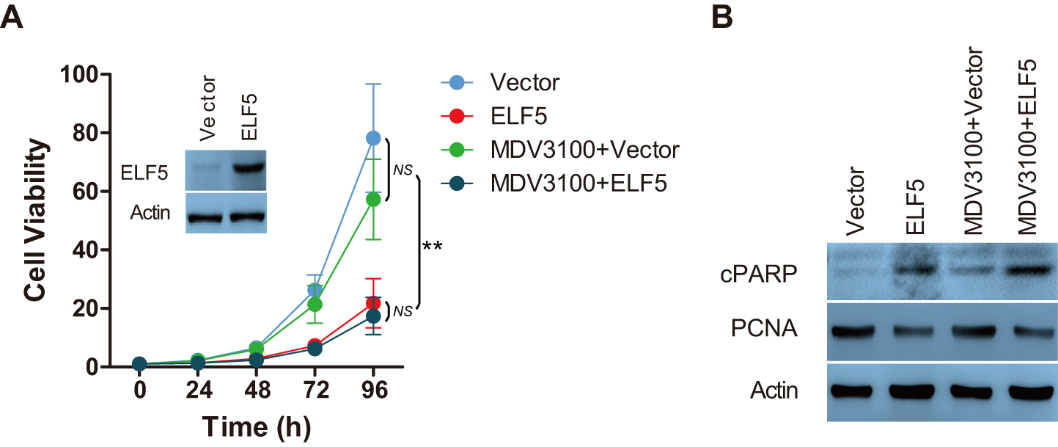
**Figure S2.** ELF5 Weaken Resistance of Prostate Cancer to Enzalutamide. (**A**) Cell growth analysis of PC3 cells with or without ELF5 overexpression followed by DMSO or enzalutamide treatment. ***P*<0.01. (**B**) WB analysis of cPARP and PCNA proteins obtained from PC3 cells with or without ELF5 overexpression followed by DMSO or enzalutamide treatment.


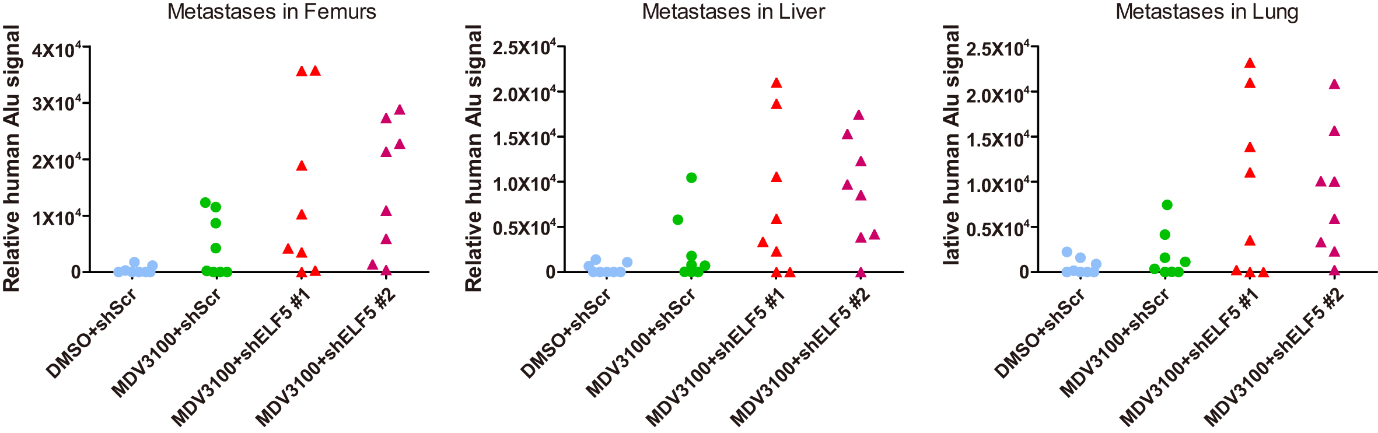


**Figure S3.** ELF5 knockdown leads to additional metastases in xenografts received enzalutamide treatment. Mice bearing xenografts arose from LNCaP cells infected by shScr or shELF5 (see also **Figure 3D**) were assessed for spontaneous metastasis to bone (femur), lung and liver after enzalutamide treatment. Results show ELF5 knockdown increases spontaneous metastasis to femur, lung and liver independent of enzalutamide treatment.


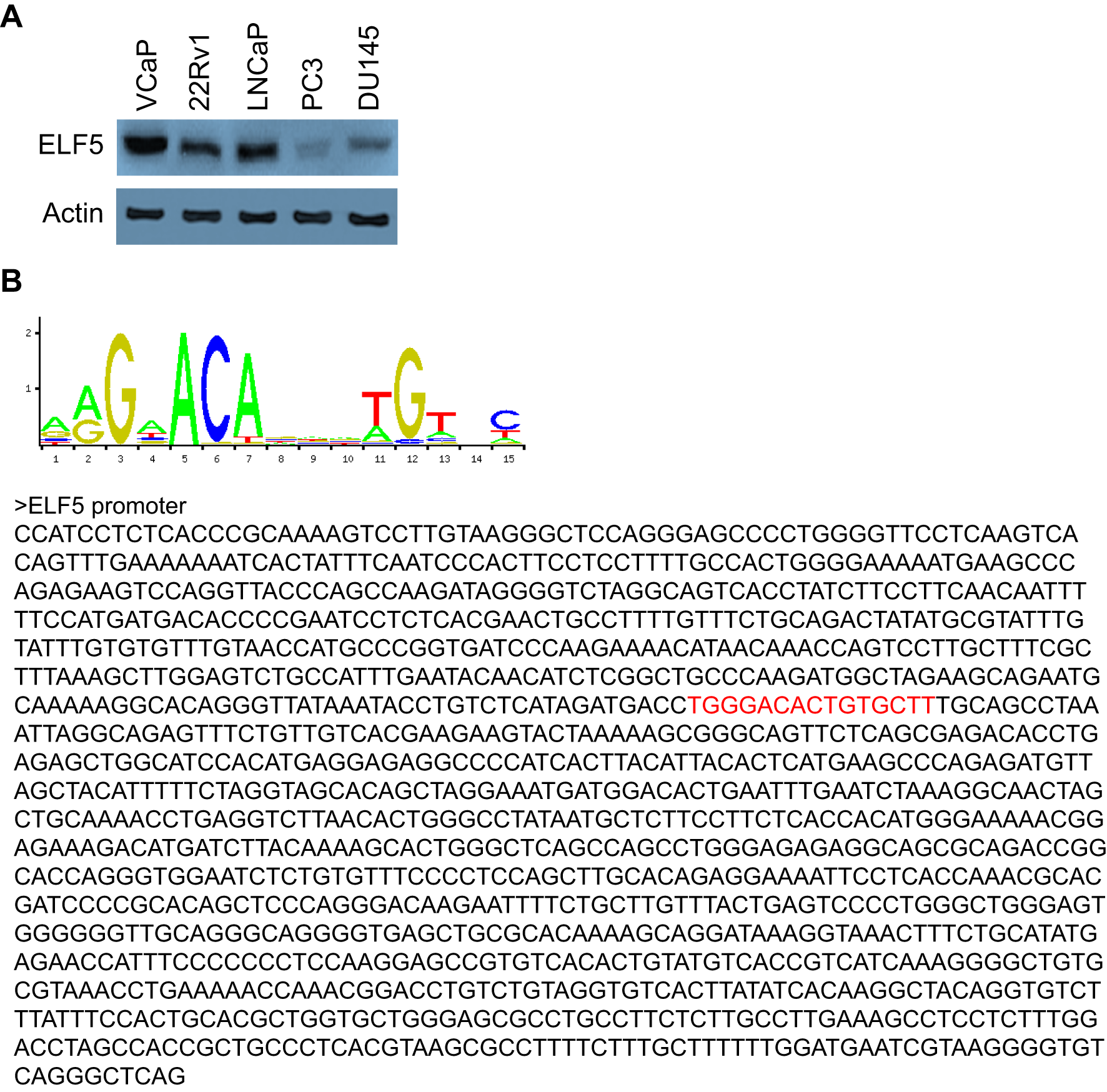


**Figure S4.** (A) WB analysis of ELF5 protein in various prostate cancer cell lines. (**B**) Bioinformatic search for AREs in ELF5 promoter. **Top**, ARE motif is shown. **Bottom**, Red Font shows canonical ARE in the ELF5 gene promoter.
